# Tracking dendritic cell migration into lymph nodes by using a novel PET probe ^18^F-tetrafluoroborate for sodium/iodide symporter

**DOI:** 10.1186/s13550-017-0280-5

**Published:** 2017-04-04

**Authors:** Sang Bong Lee, Ho Won Lee, Hongje Lee, Yong Hyun Jeon, Sang-Woo Lee, Byeong-Cheol Ahn, Jaetae Lee, Shin Young Jeong

**Affiliations:** 1 Department of Nuclear Medicine, Kyungpook National University School of Medicine and Hospital, 130 Dongdeok-ro, Jung-gu, Daegu, 41944 Republic of Korea; 2 Leading-edge Research Center for Drug Discovery and Development for Diabetes and Metabolic Disease, Kyungpook National University Medical Center, 807 Hogukro, Buk-gu, Daegu, 41404 Republic of Korea; 3 Department of Nuclear Medicine, Dongnam Institution of Radiological and Medical Sciences, 40, Jwadong-gil, Jangan-eup, Gijang-gun, Busan, 46033 Republic of Korea; 4 Laboratory Animal Center, Daegu-Gyeongbuk Medical Innovation Foundation (DGMIF), 80 Cheombok-ro, Dong-gu, Daegu, 41061 Republic of Korea; 5 Daegu-Gyeongbuk Medical Innovation Foundation (DGMIF), 80 Cheombok-ro, Dong-gu, Daegu, 41061 Republic of Korea

**Keywords:** ^18^F-tetrafluoroborate, Sodium/iodide symporter, Dendritic cell, Immunotherapy

## Abstract

**Background:**

Recently, ^18^F-tetrafluoroborate (TFB) was used as a substrate for the human sodium/iodide symporter (hNIS) reporter gene. This study evaluated the feasibility of performing molecular-genetic imaging by using the new radiotracer (^18^F-TFB) for the hNIS gene, to track dendritic cell (DC) migration in live mice. A murine dendritic cell line (DC2.4) co-expressing the hNIS and effluc genes (DC/NF) was established. To confirm the functional cellular expression of both effluc and NIS in the inoculated DC/NF cells by bio-medical imaging, combined bioluminescence imaging (BLI) and ^18^F-TFB positron emission tomography/computed tomography (PET/CT) imaging was performed after intramuscular injection with parental DCs and DC/NF cells. For DC-tracking, parental DCs or DC/NF cells were injected in the left or right mouse footpad, respectively, and ^18^F-TFB PET/CT and BLI were performed to monitor these cells in live mice.

**Results:**

In vivo PET/CT and BLI showed a clear signal in DC/NF injection sites but not in parental DC injection sites. The signal intensity in DC/NF cells was correlated with time. In vivo ^18^F-TFB PET/CT imaging showed higher radiotracer activity in the draining popliteal lymph nodes (DPLNs) in DC/NF injection sites than those in DC injection sites on day 2. BLI also showed DC/NF cell migration to the DPLNs on day 2 after the injection.

**Conclusions:**

Migration of DCs to the lymph nodes was successfully monitored using ^18^F-TFB PET/CT imaging of the NIS gene and optical imaging of the effluc gene in live mice. These data support the feasibility of using ^18^F-TFB as a substrate for hNIS reporter gene imaging to track the migration of DCs to the lymph nodes in live animals. The use of ^18^F-TFB may facilitate enhanced PET imaging of the hNIS reporter gene in small animals and humans in future studies.

**Electronic supplementary material:**

The online version of this article (doi:10.1186/s13550-017-0280-5) contains supplementary material, which is available to authorized users.

## Background

Tumor antigen-loaded dendritic cells (DCs) are used as a cancer vaccine to prevent tumors development and to eradicate existing tumors. DCs strongly express histocompatibility molecules (MHC class I and II molecules), co-stimulatory molecules (B7), and adhesion molecules (ICAM-1, ICAM-3, and LFA-3). Antigen-presenting DCs stimulate helper and killer T cells in vivo [1–3]. Antigen-specific cytotoxic CD8+ T-cells induced by DC-targeted vaccines eliminate virally infected cells and cancer cells. DC-based immunotherapy employs DCs loaded with antigens such as tumor-associated antigens. DC-based immunotherapy has been successfully used for treating many aggressive cancers [4–6]. However, to be truly effective and practical, noninvasive imaging tools are needed to evaluate the efficacy of DC-based immunotherapies to accurately track the migration of DCs to lymphoid organs.

Several non-invasive imaging techniques including gamma scintigraphy with In-111 [7], magnetic resonance imaging with iron oxide magnetic nanoparticles [8, 9], and near-infrared nanoparticles [10], and reporter gene-based imaging [11, 12] have been reported for visualization the migration of DCs. Optical imaging has a high sensitivity with low background, which enables tracking the early distribution of infused DCs. However, use of optical imaging techniques in large animals is difficult because of depth limitations. Nuclear medicine imaging techniques such as positron emission tomography (PET) provide high sensitivity without depth limitation, and can allow quantitative analysis of data for pharmacodynamics/pharmacokinetics studies. However, they have low spatial resolution and concern about radiation safety [13, 14]. I-124 has been used for PET tracer for human sodium/iodide symporter (hNIS) gene. Previously, we successfully monitored the migration of DCs to lymphoid organs in live mice using I-124 PET/computed tomography (PET/CT) imaging of hNIS gene in conjunction with optical imaging of the effluc gene [15].

In a recent study, Jauregui-Osoro et al. labeled ^18^F-tetrafluoroborate (TFB) by using current ^18^F-fluoride production and purification methods [16]. They demonstrated ^18^F-TFB uptake in normal tissues expressing NIS such as the thyroid, stomach, and salivary glands, and the inhibition of ^18^F-TFB uptake by perchlorate. They also showed that ^18^F-TFB was an effective hNIS probe in hNIS-transfected colon carcinoma cell line, HCT116 [17].

In the present study, we investigated the feasibility of performing molecular-genetic imaging with the new radiotracer ^18^F-TFB for the hNIS gene in live mice. DC2.4, an immortalized DC cell line [18], has been well characterized and is widely used for immunologic research [19, 20]. For visualizing DCs migration, DC2.4 cells that simultaneously express a dual reporter gene system were established and used for both optical and nuclear molecular imaging. Bioluminescence imaging (BLI) and ^18^F-TFB PET/CT imaging were performed to assess the initial distribution and subsequent migration of infused DCs toward the draining lymph nodes in live mice.

## Methods

### Cell line

The murine dendritic cell line, DC2.4, was cultured in RPMI1640 medium supplemented with 10% fetal bovine serum (Hyclone, Logan, UT), 0.05 mM β-mercaptoethanol (Sigma-Aldrich, St. Louis, MO), 1% non-essential amino acids (Gibco, Grand Island, NY), and 1% penicillin-streptomycin (Gibco) at 37 °C in 5% CO_2_ atmosphere. DC2.4 cells were retrovirally transduced to express both the effluc and Thy1.1 genes [15]. Thy1.1-positive cells were sorted using CD90.1 microbeads (Miltenyi Biotec, Bergisch Gladbach, Germany). DC2.4 cells expressing the effluc gene were then retrovirally transduced to express both the hNIS and enhanced green fluorescent protein (EGFP) genes. After staining the cells with an APC-Cy7-conjugated anti-Thy1.1 antibody (BD Biosciences), Thy1.1+/- EGFP+ cell population was enriched using FACS Aria III (BD Biosciences). The established stable cells co-expressing effluc and hNIS were referred to as DC/NF cells.

### ^18^F-TFB synthesis

^18^F-TFB was synthesized using a labeling protocol developed previously [16]. Briefly, ^18^F-fluoride (12–18 GBq) trapped in a QMA cartridge was eluted using 1.5 mol/L HCl into a reactor containing sodium tetrafluoroborate. The reaction mixture was heated to 120 °C for 10 min, cooled to 25 °C, passed through a silver ion-loaded cation exchange cartridge and two Sep-Pak Light Alumina N, and sterile filtered.

### ^18^F-TFB analytical methods

Labeling was monitored by performing thin-layer chromatography (TLC) with alumina TLC strips, fluorescent indicator (length, 100 mm; Sigma-Aldrich, Gillingham, UK), and methanol as the mobile phase. The strips were scanned using Bio-scan 2000 radio TLC scanner. Presence of TFB at Rf: 0.60–0.65 was determined by scraping alumina from the TLC plate.

### ^18^F-TFB uptake assay

Indicated number of cells was plated in 24-well plates. After 1 day, the cells were incubated with 500 μL Hank’s balanced salt solution (HBSS) containing 0.5% bovine serum albumin (bHBSS), 3.7 kBq carrier-free ^18^F-TFB, and 10 μmol/L sodium iodide (specific activity 740 MBq/mmol) at 37 °C for 30 min. After incubation, the cells were washed twice with ice-cold bHBSS buffer as quickly as possible and were lysed using 500 μL 2% SDS. Radioactivity was measured using Packard Cobra II gamma-counter (PerkinElmer, Waltham, MA).

### Cell proliferation assay

Cell proliferation was determined using cell counting kit (CCK)-8 (Dojindo Laboratories, Tokyo, Japan). Briefly, parental DC2.4 cells and DC/NF cells were plated in 96-well plates at a density of 1 × 10^4^ cells/well. After 1 day, 10 μL CCK-8 solution was added to each well, and the plates were incubated at 37 °C for 3 h. Absorbance was measured at 450 nm by using BMG Lab Tech microplate reader.

### Phenotypic analysis

Parental DC2.4 and DC/NF cells were stained with PE-conjugated CD54, CD86, H-2Kb (MHC class I), and I-A/I-E (MHC class II), and APC-conjugated CD205 (DEC-205) at 4 °C for 30 min. The cells were washed twice with 0.1% BSA/PBS and analyzed using FACS Accuri C6 flow cytometer (BD Biosciences). Isotype-matched monoclonal antibodies were used as controls.

### In vivo imaging (PET/CT imaging and BLI)

**Fig. 1:**
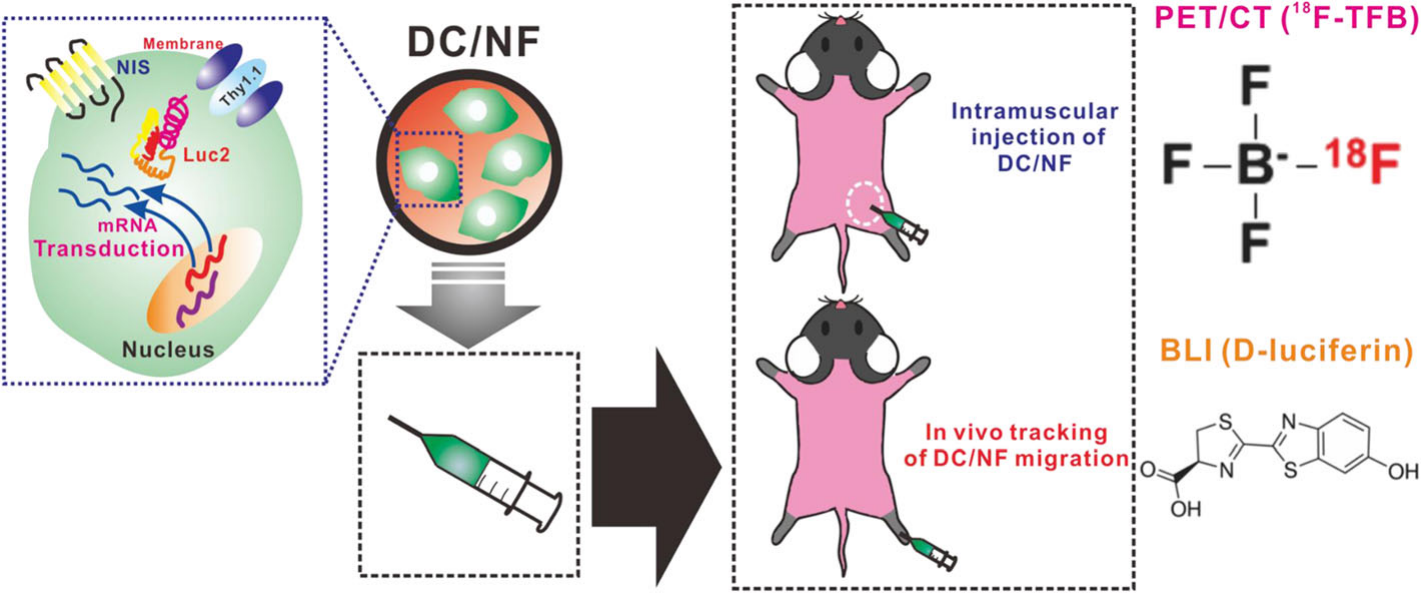
Schematic representation of the in vivo tracking of DC migration by performing multimodal reporter gene imaging. The multimodal reporter system involves the NIS, Luc2, and Thy1.1 genes, which act as nuclear reporter, optical reporter, and surrogate for the NIS and Luc2 genes, respectively. The multimodal reporter genes were introduced into DCs retrovirally, and the reporter-tagged DCs were injected into the muscles and footpads of mice. Next, combined PET/CT imaging (^18^F-TFB) and BLI (d-luciferin) was performed
- *Study 3*: Migration of DCs toward lymphoid organs was monitored by performing animal experiments, as depicted in Fig. 1 and Additional file 1: Figure S2. Parental DC2.4 cells and DC2.4/NF cells (1.5 × 10^6^ DCs/mouse) were subcutaneously injected into the left and right hind footpads of mice (*n* = 5), respectively, and BLI and ^18^F-TFB PET/CT images were obtained on day 2 after the injection.

- *Study 1*: ^18^F-TFB was injected into the via tail vein of mice (*n* = 5) and PET/CT images were acquired at 30 min after the injection.
- *Study 2*: Animal experiments were conducted as depicted in Fig. 1 and Additional file 1: Figure S1. Parental DC and DC/NF cells (1 × 10^7^ each) were intramuscularly injected into the left and right hind thighs of mice (*n* = 5), respectively. BLI and ^18^F-TFB PET/CT imaging were performed on days 1 and 4 after the injection.

For PET/CT imaging, a 20 min (tumor imaging) scan was performed using the Triumph II PET/CT system (LabPET8, Gamma Medica-ideas, Waukesha, WI). CT scans were performed using an X-ray detector (fly acquisition; number of projections, 512; binning setting, 2 × 2; frame number, 1; X-ray tube voltage, 75 kVp; focal spot size, 50 μm; magnification factor, 1.5; and matrix size, 512) immediately after acquisition of the PET images. The PET images were reconstructed using 3D-OSEM iterative image reconstruction, and the CT images were reconstructed using filtered back-projections. All mice were anesthetized using 1–2% isoflurane gas during imaging. The PET images were co-registered with anatomical CT images by using a 3D image visualization and analysis software VIVID (Gamma Medica-ideas, Northridge, CA). To measure uptake (counts) for volumes of interest (VOIs), VOIs from each image were manually segmented from co-registered CT images by using VIVID and PMOD software (PMOD Technologies, Zurich, Switzerland). Uptake in the region of interest (ROI) was measured using PMOD 3.5 software.

BLI was performed using IVIS Lumina III imaging system (PerkinElmer) 10 min after an intraperitoneal injection of d-luciferin (3 mg/mouse; PerkinElmer). Grayscale photographic images and bioluminescent color images were superimposed using LIVINGIMAGE software (version 2.12; PerkinElmer) and IGOR Image Analysis FX software (WaveMetrics, Lake Oswego, OR, USA). Bioluminescent signals are expressed as photons per cm^2^ per second per steradian (P/cm^2^/s/sr).

### Statistical analysis

All data are expressed as mean ± standard deviation, of at least three independent experiments. Statistical significance was determined using an unpaired Student’s *t* test, with Graph Pad Prism version 5 (GraphPad software Inc.). Differences with *p* values less than 0.05 were considered statistically significant.

## Results

### Synthesis and characterization of ^18^F-TFB

Previously, Jauregui-Osoro et al successfully established ^18^F-TFB, an hNIS imaging agent, as a novel imaging agent for PET/CT imaging (Additional file 1: Figure S3A) [16]. Fluorination reaction was performed by using a Na^18^F labeling TFB solution. The reaction was completed within 20 min as monitored using the radio-TLC scanner (eluent: methanol). After reaction completion, the solution was column filtrated for Na^18^F to remove the unreacted radioactive Na^18^F. After purification, radiochemical purity of ^18^F-TFB was found to be over 90% (Additional file 1: Figure S3B).

### NIS gene uptake and in vivo PET imaging of intravenously injected ^18^F-TFB in normal mice

**Fig. 2:**
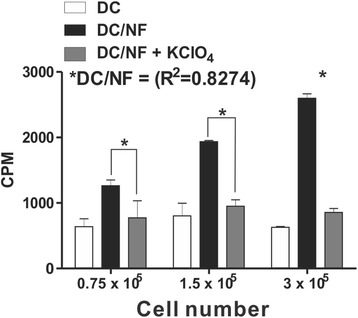
In vitro ^18^F-TFB uptake into DC and DC/NF cells. Data are means ± S.D., *n* = 5

Rapid accumulation of ^18^F-TFB was observed in hNIS-expressing DCs (DC/NF cells). ^18^F-TFB uptake increased in a cell number-dependent manner in DC/NF cells, but not in parental DCs (Fig. 2). Uptake in DC/NF cells was dramatically inhibited by potassium perchlorate (KClO_4_) a well-established specific inhibitor of ^18^F-TFB uptake by NIS. In DC/NF cells, ^18^F-TFB uptake was 4.1-fold and 3.2-fold-higher than that in parental DCs and KClO_4_-treated DC/NF cells, respectively (*R* ^2^ = 0.8274).

Next, we monitored the in vivo distribution of intravenously injected ^18^F-TFB in mice to demonstrate the imaging agents. Mice received ^18^F-TFB via tail vein injection, and PET imaging was performed at 30 min after the injection. Uptake of ^18^F-TFB was observed NIS-expressing organs such as the thyroid and stomach at 30 min after the injection.

### Effect of hNIS gene transduction on the function of DCs

**Fig. 3:**
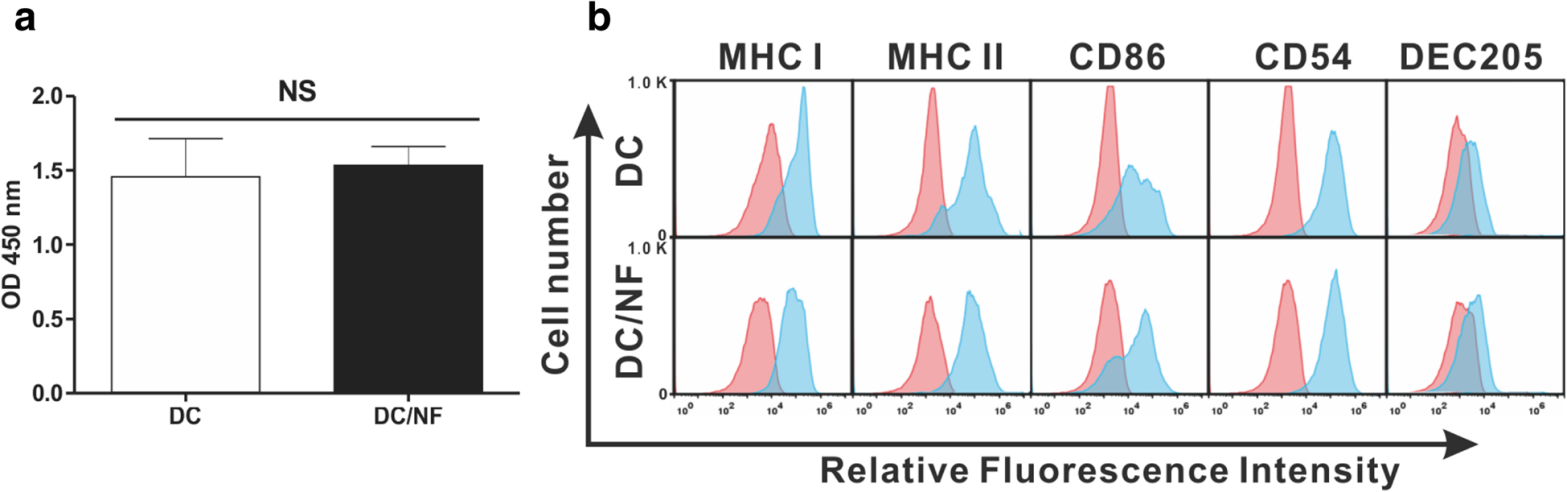
Effect of hNIS transduction on DC function. **a** Cell proliferation rates of parental DC and DC/NF cells. No significant difference was observed in the proliferation rate of the two cell lines. **b** Phenotypic analysis of parental DC and DC/NF cells. Cells were stained with PE-conjugated CD54, CD86, and H-2Kb (MHC class I) and I-A/I-E (MHC class II) and APC-conjugated CD205 (DEC-205), respectively. *Red* histograms represent isotype control. Data are expressed as mean ± SD; *n* = 5

The effects of cellular labeling by virus infection in the immune cells confirm the cell proliferation and phenotype marker expression levels in parental DCs and DC/NF cells. The cell viability assay (CCK-8) indicated no significant difference in the proliferation of parental DCs and DC/NF cells (Fig. 3a). Moreover, phenotype marker analysis showed that the expression of DC-specific markers such as MHC class I and II, CD86, CD54, and DEC205 was not different between parental DCs and DC/NF cells (Fig. 3b).

### In vivo ^18^F-TFB PET/CT imaging and BLI of DC and DC/NF cells in mice

**Fig. 4:**
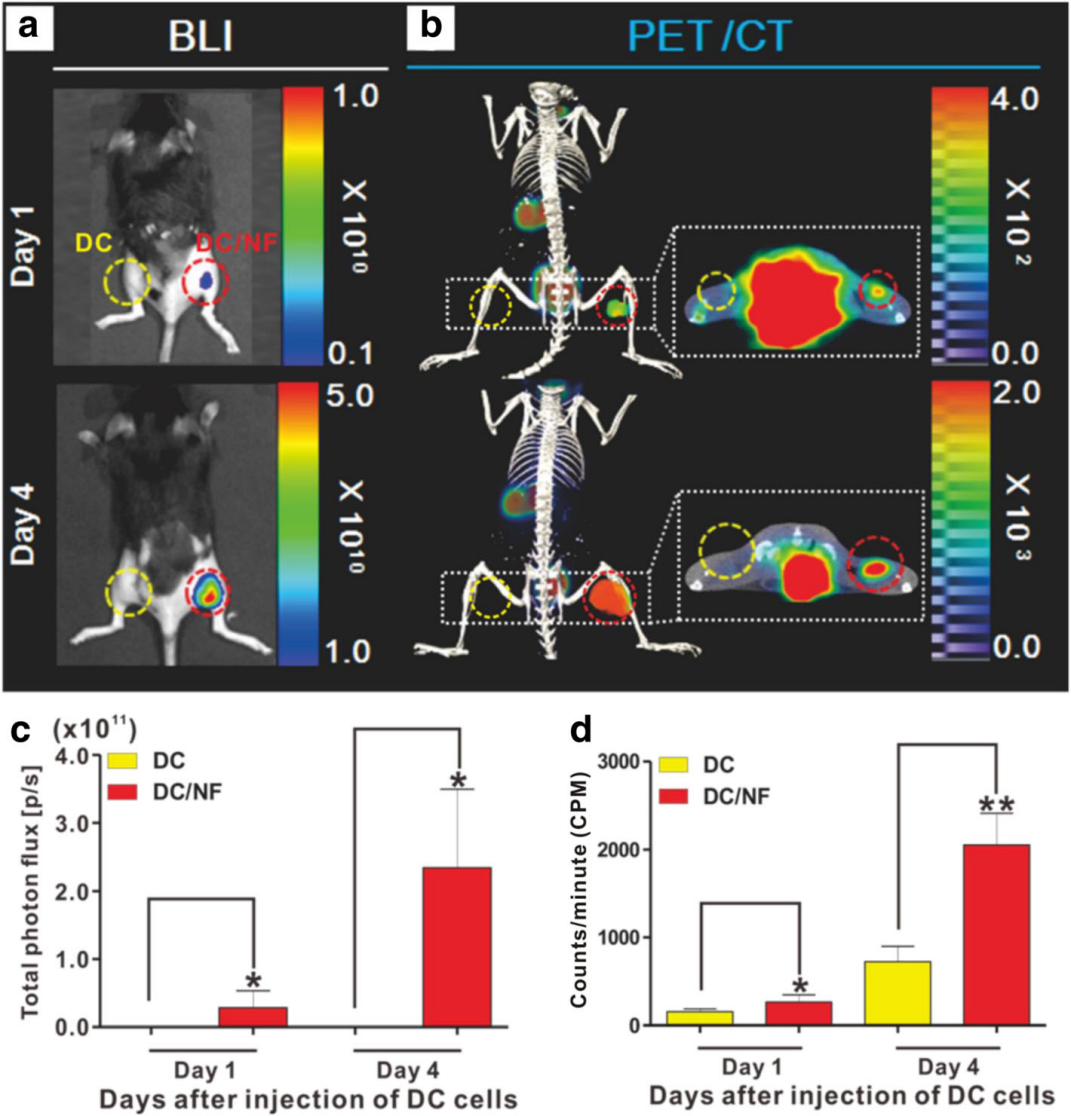
In vivo ^18^F-TFB PET/CT imaging and BLI of intramuscularly injected DCs. Parental DC and DC/NF cells were intramuscularly injected into the left and right thighs of mice, respectively, and combined ^18^F-TFB PET/CT imaging and BLI was performed. **a** In vivo BLI and **b** ^18^F-TFB PET/CT imaging of parental DC and DC/NF cells on days 1 and 4 after the intramuscular injection. The *red* and *yellow circles* indicate the DC and DC/NF injection sites, respectively. ROI analysis of **c** bioluminescent and **d** PET/CT images. Data are expressed as mean ± SD; *n* = 5

Combined BLI and ^18^F-TFB PET/CT imaging was performed after the intra-muscular injection of parental DCs and DC/NF cells to confirm the functional cellular expression of both effluc and NIS in the inoculated DC/NF cells by bio-medical imaging (Fig. 4a, b). In vivo BLI and PET/CT showed an evident signal in DC/NF injection sites, but not in parental DC injection site. Moreover, BLI and PET/CT images clearly demonstrate DC/NF uptake in the right hind thigh on days 1 to 4. Signal intensity in DC/NF cells was correlated with time. Quantification showed a time-dependent correlation between bioluminescence signals and ^18^F-TFB uptake (Fig. 4c, d). Bioluminescence signal within the DC/NF injection site on day 4 was 8.3-fold higher than that on day 1, and ^18^F-TFB uptake within the DC/NF injection site on day 4 was 7.2-fold higher than that on day 1.

### In vivo imaging of DC migration by the hNIS and effluc genes in live mice

**Fig. 5:**
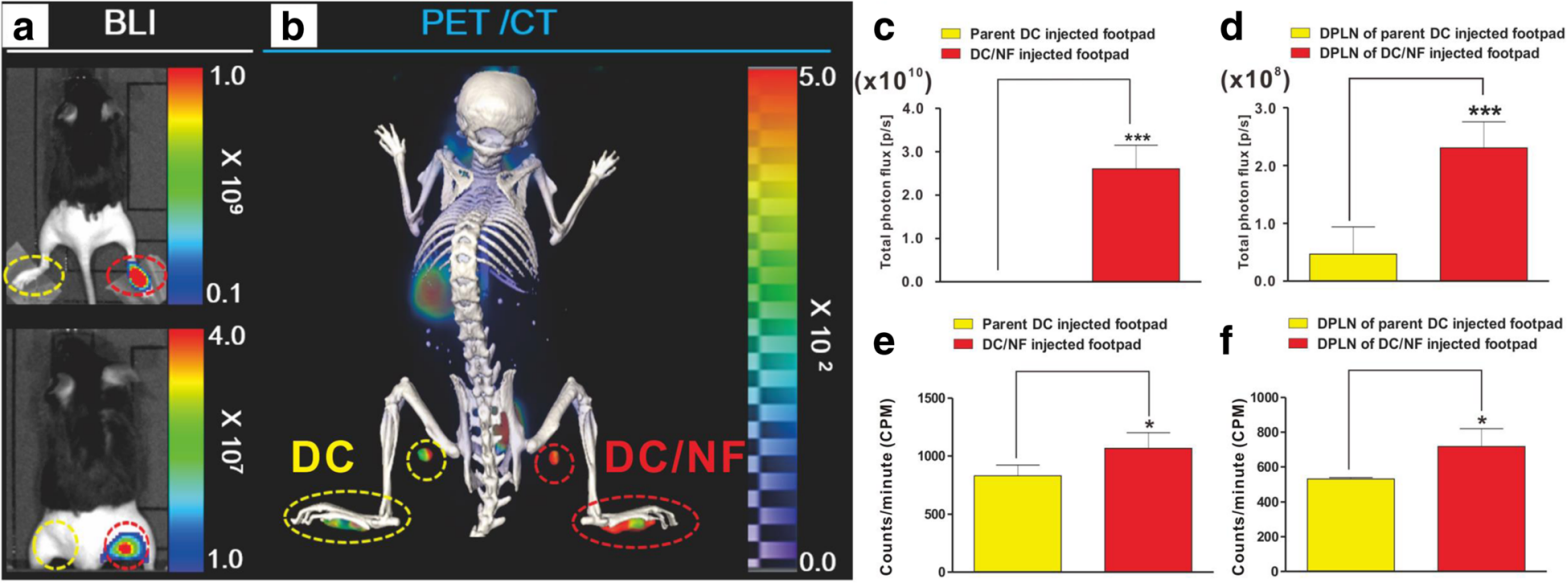
In vivo combined BLI and ^18^F-TFB PET/CT imaging of DC migration after their subcutaneous injection into footpads. **a** In vivo BLI (*upper panel*: without masking the footpads; *lower panel*: after masking the footpads) and **b** ^18^F-TFB PET/CT imaging of DC/NF cell migration to the DPLNs after their injection into mouse footpads. Quantitative analysis of **c**, **d** bioluminescence signals (P/cm2/s/sr) and **e**, **f** radioactivity (count per minute) in DC-injected footpads and DPLNs. The *red* and *yellow circles* indicate the DC and DC/NF injection sites and DPLNs, respectively. Data are expressed as mean ± SD; *n* = 5

To monitor the migration of DC/NF cells toward draining popliteal lymph nodes (DPLNs), parental DC and DC/NF cells were subcutaneously injected in to the respective footpads, as described in Additional file 1: Figure S2. BLI of d-luciferin were obtained on day 2 after injection of DCs. Strong bioluminescence signals were detected in the DC/NF cell-injected footpads (upper figure of Fig. 5a). However, the DPLNs of DC/NF cell-injected footpads did not show any BLI signals because the DC/NF cell-injected footpad has strong BLI signals. In vivo BLI was performed after masking the DC/NF footpads to allow the detection of distinct signals in DPLNs. Migrated DC/NF cells were clearly observed in DPLNs of DC/NF cell-injected footpads (bottom of Fig. 5a). Quantification analysis of bioluminescence signals in the DC-injected footpads (1), DPLNs of DC-injected footpads (3), DC/NF cell-injected footpads (2), and DPLNs of DC/NF cell-injected footpads (4) were 1.3 × 10^8^ ± 1.8 × 10^7^, 4.6 × 10^7^ ± 4.7 × 10^6^, 2.3 × 10^10^ ± 6.2 × 10^9^, and 2.3 × 10^8^ ± 4.5 × 10^7^ P/cm2/s/sr, respectively (Fig. 5c, d).

^18^F-TFB uptake was more intense in DC/NF cell-injected footpads and DPLNs of DC/NF cell-injected footpads than in parental DC-injected footpads and DPLNs of paternal DC-injected footpads (Fig. 5b), which was consistent with the results of BLI. Uptake values of DC/NF cell-injected footpads, DPLNs of DC/NF cell-injected footpads, and DC-injected footpads and DPLNs of DC-injected footpads were 829.7 ± 92.2, 530.9 ± 7.84, 1066.6 ± 135.4, and 718.2 ± 102.3 counts, respectively (Fig. 5e, f).

## Discussion

Recent studies have investigated the hNIS gene as a reporter gene in gene therapy and for imaging cell migration and differentiation [21–23]. TFB is a fluorine-containing ion that interacts with hNIS. Anbar et al. suggested that labeled TFB has a good potential for thyroid imaging [24–26] because TFB inhibits iodide uptake in the thyroid in vivo and specifically accumulates in the thyroid [25, 27]. Jauregui-Osoro et al. established ^18^F-TFB as a new PET imaging agent for hNIS. They demonstrated that ^18^F-TFB rapidly accumulated in a thyroid cell line that was stimulated by the thyroid-stimulating hormone and that ^18^F-TFB uptake was inhibited by perchlorate. Rapid specific accumulation of ^18^F-TFB was also observed in NIS-expressing normal tissues such as the thyroid, stomach, and the salivary glands and this accumulation was also inhibited by perchlorate [16]. In the present study, we successfully developed ^18^F-TFB by using an isotopic exchange methods. The radiochemical purity of ^18^F-TFB exceeded 90%, which was consistent with that reported in a previous study. In the present study, in vitro ^18^F-TFB uptake increased in NIS expressing cells and was inhibited by KClO_4_, a well-established specific inhibitor of iodide uptake by hNIS. Furthermore, ^18^F-TFB biodistribution in normal mice was similar to the physiological distribution of NIS. PET/CT imaging by using ^18^F-TFB clearly differentiated among the thyroid tissue, gastric mucosa, and bladder, with a high signal-to-noise ratio. These results are consistent with those of a previous study showing normal distribution of ^18^F-TFB [16].

Traditionally, radioiodides ([^131^I]iodine and [^123^I]iodine) and ^99m^Tc-pertechnetate, which emit γ-rays, have been used for imaging endogenous and exogenous hNIS. However, imaging with these radioiodides is associated with limitations such as reduced resolution and relatively long half-life of these radionuclides. Single-photon emission computed tomography (SPECT) by using ^99m^Tc-pertechnetate was used for the in vivo tracking of NIS-expressing cell. Fruhwirth et al. detected NIS-expressing tumor cells by performing SPECT with ^99m^Tc-pertechnetate in a preclinical metastasis model [28]. SPECT by using ^99m^Tc-pertechnetate provides high-resolution images. However, PET imaging offers better image resolution and detection sensitivity than SPECT. Furthermore, PET imaging allows quantitative analysis. Recent studies have used ^124^I for dose planning in radionuclide therapy and immune cell tracking studies [15, 29–31]. However, PET imaging of NIS by using ^124^I also is associated with some drawbacks. PET imaging with ^124^I is not as efficient as expected, because of its complex decay scheme, with a low abundance of positrons (23%) and emission of high-energy γ photons. The high-energy γ photons have an energy close to 511 keV energy window and compete in abundance with positron decay (Emax = 1.69 MeV, 10%). These may interfere with annihilation photons leading to increased background noise and poor image quality. Moreover, ^124^I has a long half-life (4.18 days), which cause a high absorbed dose of radiation and availability of ^124^I is limited due to its complex production.

In contrast, an ideal PET tracer would be based on ^18^F because ^18^F has good imaging characteristics such as appropriate half-life (110 min), high positron yield (97%), low positron energy (Emax = 0.634 MeV), and easy accessibility due to production using a medical cyclotron. The utility of the new radiotracer, ^18^F-TFB, to visualize the thyroid and other NIS-expressing organs has been previously described in a preclinical study assessing PET/CT images of rodents [16]. Thus, our results indicate that nuclear medicine imaging with the hNIS gene is a feasible technique for tracking the migration of DCs to lymphoid organs in live mice. By performing ^18^F-TFB PET/CT imaging, we successfully demonstrated the proliferation of DC/NF cells in mice from days 1 to 4 after their intramuscular injection. Furthermore, in vivo ^18^F-TFB PET/CT imaging as well as BLI showed the migration of DCs to DPLNs of DC/NF cell-injected footpads on day 2 after their injection. Results of ^18^F-TFB PET/CT imaging obtained in the present study are similar to those obtained of ^124^I PET/CT imaging for tracking DC/NF cells in our previous study [15]. However, because of the high signal-to-noise ratio, visualization of migrated DCs in DPLNs was better in ^18^F-TFB PET/CT images than in ^124^I PET/CT images. These results first demonstrated the use of ^18^F-TFB PET/CT for immune cells in addition to thyroid tissues and exogenous cancer cell expression, suggesting that NIS combined with ^18^F-TFB PET imaging is a good tool for cell tracking.

In the present study, we demonstrated that nuclear medicine imaging with the NIS gene is a feasible technique for tracking the migration of DCs to lymphoid organs in live mice. Because in vivo imaging with the NIS gene is hampered by both high background noise and signals emitted from normal tissues and non-specific organs, it is difficult to visualize the initial distribution and low population of infused DCs. To overcome these hurdles for tracking DCs with the NIS gene, we combined in vivo nuclear medicine imaging with BLI based on the effluc gene, which allows tracking the early distribution of DCs and verifying the feasibility of the NIS gene as a reporter for tracking the migration of DCs to lymphoid organs. Thus, an effluc reporter gene was additionally adopted for tracking the migration of DCs and dual reporter DCs expressing both the NIS and effluc genes were successfully established using a viral vector system expressing the reporter genes.

In vivo DC migration efficiency is unsatisfactory (typical rate ≤ 4%) when DCs are administered through an intradermal injection, which is the most common method used in DC-based immunotherapy. In the present study, in vivo DC migration efficiency was approximately 1% on day 2. The most commonly used method to improve the migration of DCs is the pre-injection of pro-inflammatory cytokines that helps provide a suitable inflammatory microenvironment for the migration of DCs through lymphatic vessels [32]. Pre-injection of mouse tumor necrosis factor (TNF) increased the number of DCs reaching the LNs by approximately 10-fold [33]. Alternatively, increasing the expression levels of specific DC-homing receptors also facilitates their migration [34]. For example, DCs adenovirally transduced with the C-C motif chemokine receptor 7 gene, which encodes a chemokine receptor responsible for the migration of DCs toward lymphatics, increases the migration efficiency of DCs by approximately sixfold relative to that of control DCs [35]. Thus, DC migration monitoring using the hNIS gene and ^18^F-TFB could be a valuable approach in experiments for improving the migration efficiency and antitumor efficacy of DCs.

Although ^18^F-TFB is a promising PET imaging probe for hNIS, the ^18^F-TFB synthesis method used at present involving isotopic exchange provides ^18^F-TFB with a suboptimal radiochemical yield and specific activity. The specific activity of ^18^F-TFB was found to be approximately 1 GBq/μmol in a previous study [16], which was similar to that obtained in the present study (0.65 GBq/μmol). Recently, Khoshnevisan et al. developed a method for synthesizing ^18^F-TFB with high specific activity and sufficient yield [36]. Jiang et al also reported a new ^18^F-TFB synthesis method which was achieved via direct radiofluorination of boron trifluoride using an automated synthesis system [37]. Using these new synthesis methods, high-specific-activity synthesis of ^18^F-TFB was achieved. The increased specific activity of ^18^F-TFB may allow for enhanced PET imaging of the hNIS reporter gene in future human and animal studies.

## Conclusions

In conclusion, we successfully monitored the proliferation of DCs and their migration to lymph nodes in live mice by performing ^18^F-TFB PET imaging of the NIS gene and optical imaging of the effluc gene. Together, these results highlight the potential of ^18^F-TFB as a new NIS imaging probe for cell tracking. Further studies should be performed to use this probe for tracking other immune cells such as cytotoxic T lymphocytes and natural killer cells.

## Additional files

Additional file 1: Figure S1. Schematic diagram for the in vivo monitoring of reporter DC/NF cells after their intramuscular injection. Briefly, combined BLI and ^18^F-TFB PET/CT imaging was performed on day 1 or 4 after the injection of DC and DC/NF cells into the right and left thighs of mice, respectively. Figure S2. Schematic diagram for the in vivo monitoring of reporter DC/NF cell migration to the DPLNs. In vivo BLI and ^18^F-TFB PET/CT imaging were performed on day 2 after the injection of DC and DC/NF cells in the right and left footpads of mice, respectively. Figure S3. Synthesis and characterization of ^18^F-TFB. (A) Schematic representation of ^18^F-TFB synthesis. (B) Chromatograms of radio-TLC to monitor the radiolabeling reaction. (PPTX 1047 kb)

## Abbreviations

APC: Allophycocyanin
BLI: Bioluminescence imaging
CCK: Cell counting kit
CT: Computed tomography
DCs: Dendritic cells
DPLNs: Draining popliteal lymph nodes
EGFP: Green fluorescent protein
HBSS: Hank’s balanced salt solution
hNIS: Human sodium/iodide symporter
KClO4: Potassium perchlorate
MHC: Major histocompatibility complex
PE: Phycoerythrin
PET: Positron emission tomography
ROI: Region of interest
SPECT: Single-photon emission computed tomography
TFB: Tetrafluoroborate
TLC: Thin-layer chromatography
TNF: Tumor necrosis factor
VOIs: Volumes of interest
