# Supplementary figures and images for: Tracking dendritic cell migration into lymph nodes by using a novel PET probe 18F-tetrafluoroborate for sodium/iodide symporter
Source: EJNMMI Res. 2017 Apr 4;7:32. doi: 10.1186/s13550-017-0280-5 (PMC5380646; doi:10.1186/s13550-017-0280-5)

## Slide 1
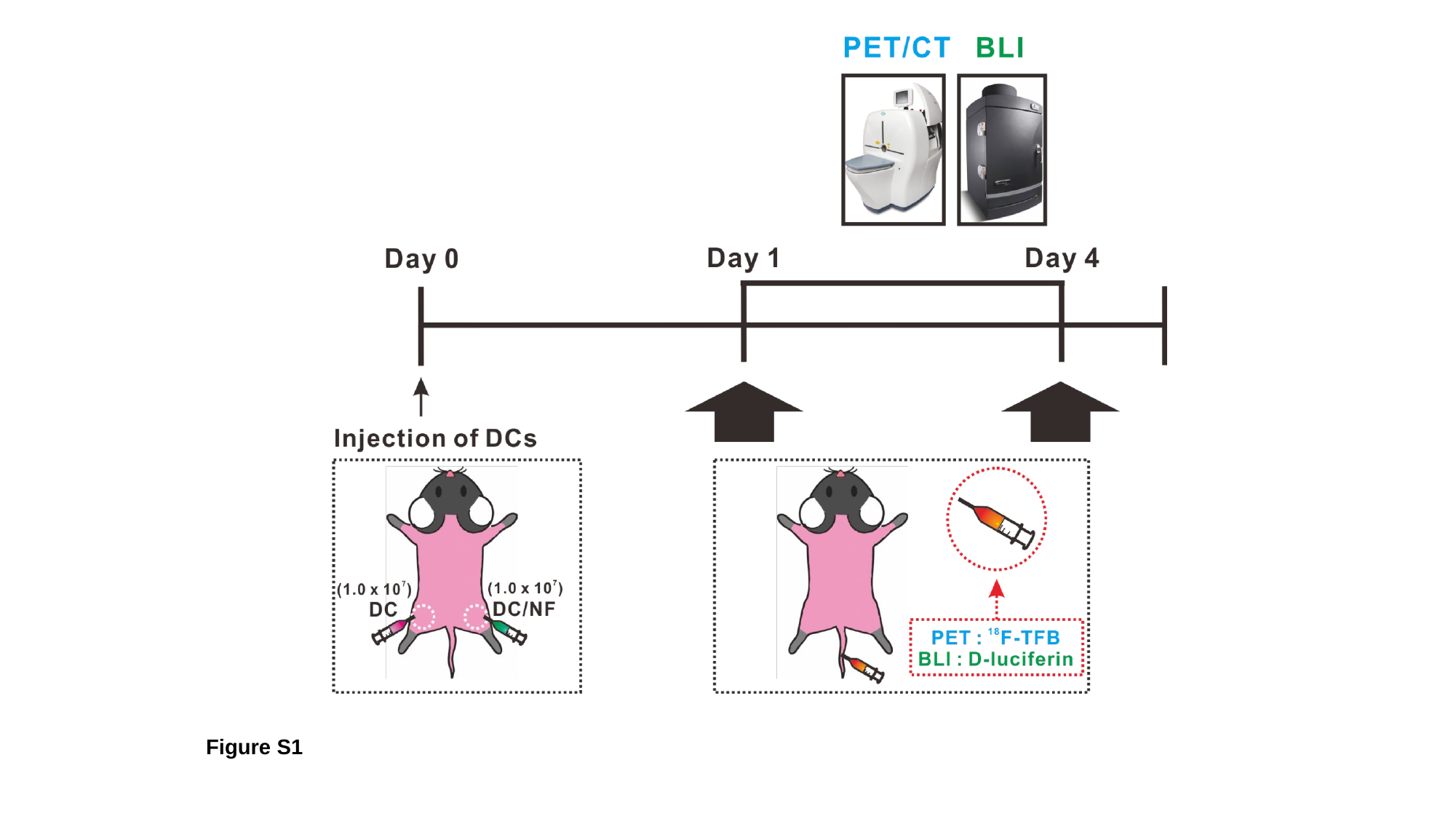

Figure S1

## Slide 2
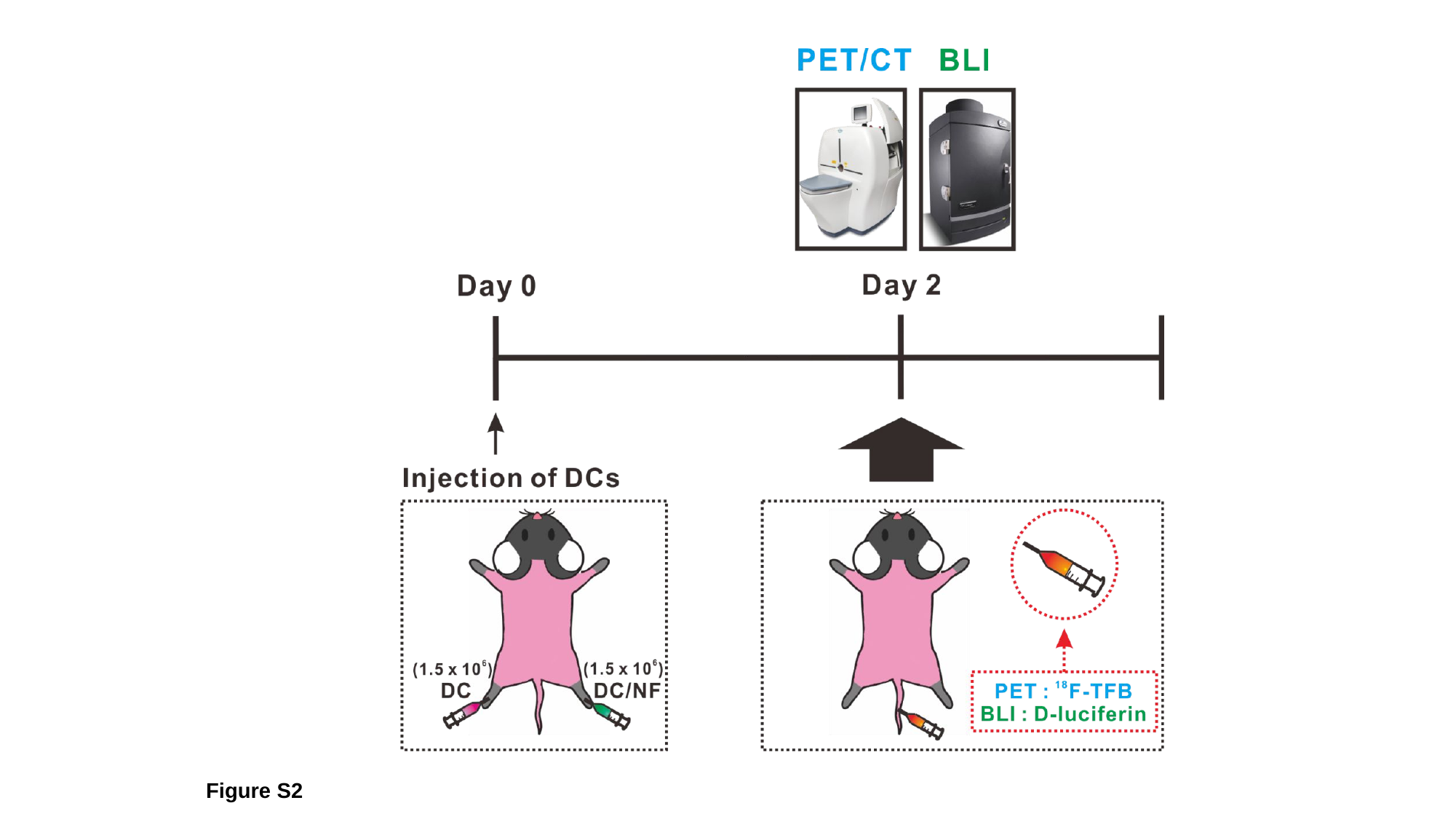

Figure S2

## Slide 3
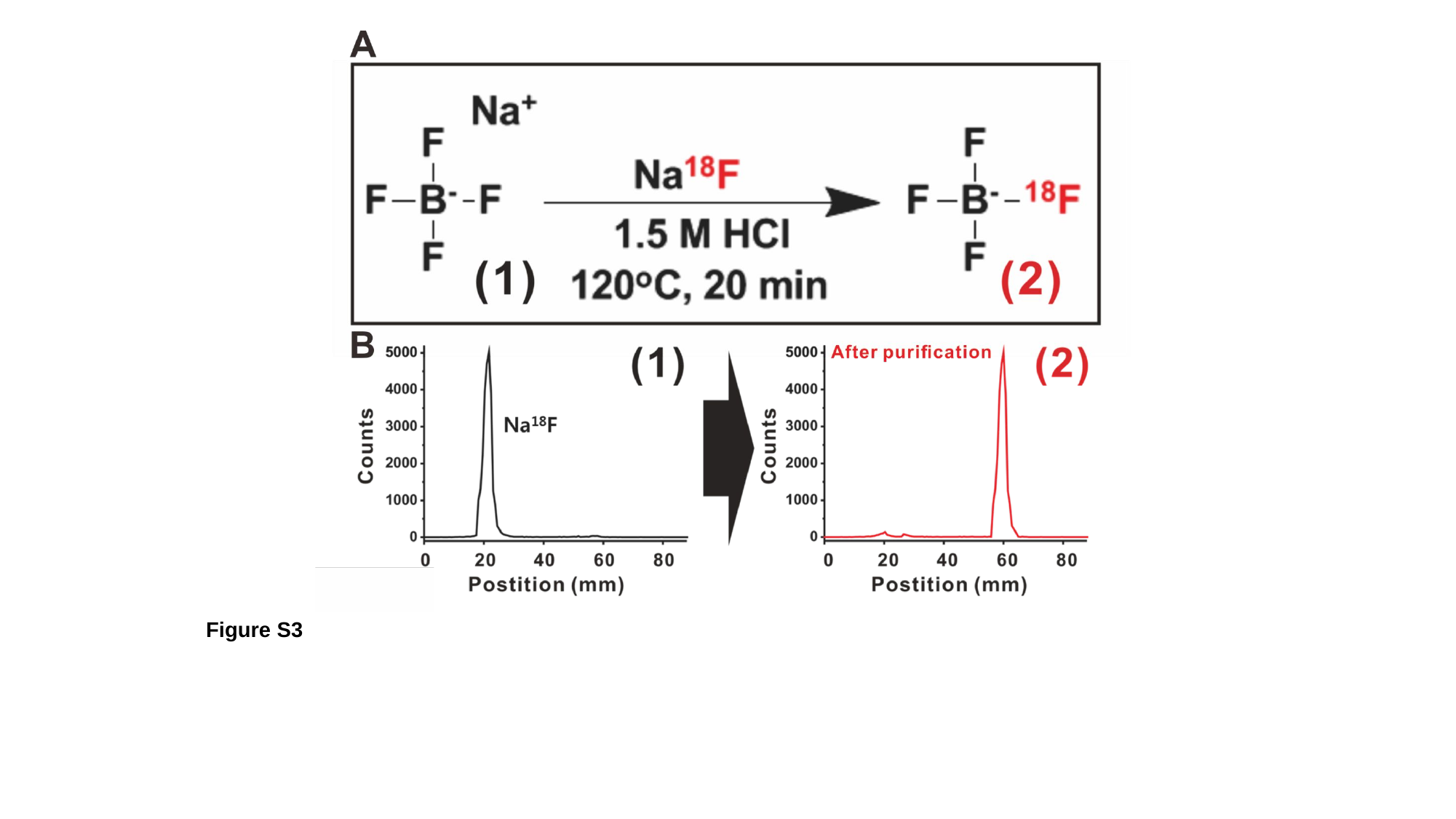

Figure S3

Supplement: Supplementary file 1 — Schematic diagram for the in vivo monitoring of reporter DC/NF cells after their intramuscular injection. Briefly, combined BLI and 18F-TFB PET/CT imaging was performed on day 1 or 4 after the injection of DC and DC/NF cells into the right and left thighs of mice, respectively. Figure S2. Schematic diagram for the in vivo monitoring of reporter DC/NF cell migration to the DPLNs. In vivo BLI and 18F-TFB PET/CT imaging were performed on day 2 after the injection of DC and DC/NF cells in the right and left footpads of mice, respectively. Figure S3. Synthesis and characterization of 18F-TFB. (A) Schematic representation of 18F-TFB synthesis. (B) Chromatograms of radio-TLC to monitor the radiolabeling reaction. (PPTX 1047 kb) [file 13550_2017_280_MOESM1_ESM.pptx]
